# Extraordinary clinical response to ibrutinib in low-grade ovarian cancer guided by organoid drug testing

**DOI:** 10.1038/s41698-023-00379-8

**Published:** 2023-05-18

**Authors:** Heidi J. Gray, Payel Chatterjee, Rachele Rosati, Lauren R. Appleyard, Grace J. Durenberger, Robert L. Diaz, Hallie A. Swan, Danielle Peretti, Maddy Pollastro, Trevor Ainge, Katannya Kapeli, Shalini Pereira, Astrid L. Margossian, Kalyan Banda, Barbara A. Goff, Elizabeth M. Swisher, Brady Bernard, Christopher J. Kemp, Carla Grandori

**Affiliations:** 1 Division of Gynecology Oncology, University of Washington, Seattle, WA USA; 2 SEngine Precision Medicine, Seattle, WA USA; 3 Clinical Research Division, Fred Hutchinson Cancer Center and Division of Medical Oncology, University of Washington, Seattle, WA USA; 4 Earle A. Chiles Research Institute, Providence Cancer Institute, Portland, OR USA; 5 Division of Human Biology, Fred Hutchinson Cancer Center, Seattle, WA USA

**Keywords:** Ovarian cancer, Ovarian cancer

## Abstract

Low-grade serous ovarian cancer (LGSOC) typically responds poorly to standard platinum-based chemotherapy and new therapeutic approaches are needed. We describe a remarkable response to targeted therapy in a patient with platinum-resistant, advanced LGSOC who had failed standard-of-care chemotherapy and two surgeries. The patient was in rapid decline and entering hospice care on home intravenous (i.v.) opioid analgesics and a malignant bowel obstruction requiring a G-tube. Genomic analysis of the patient’s tumor did not indicate obvious therapeutic options. In contrast, a CLIA-certified drug sensitivity assay of an organoid culture derived from the patient’s tumor identified several therapeutic choices, including Bruton’s tyrosine kinase (BTK) inhibitor ibrutinib, as well as the EGFR inhibitors afatinib and erlotinib. Following off-label administration of daily ibrutinib as monotherapy, the patient had an exceptional clinical turnaround over the following 65 weeks with normalization of CA-125 levels, resolution of the malignant bowel obstruction, halting of pain medications, and improvement of performance status from ECOG 3 to ECOG 1. After 65 weeks of stable disease, the patient’s CA-125 levels began to rise, at which point the patient discontinued ibrutinib and began taking afatinib as monotherapy. The patient’s CA-125 levels remained stable for an additional 38 weeks but due to anemia and rising CA-125 levels, the patient switched to erlotinib and is currently being monitored. This case highlights the clinical utility of ex vivo drug testing of patient-derived tumor organoids as a new functional precision medicine approach to identify effective personalized therapies for patients who have failed standard-of-care treatments.

## Introduction

Epithelial ovarian cancer (EOC) is the most lethal gynecological cancer. Serous ovarian cancer is the most common histologic subtype of EOC and is divided into high-grade serous ovarian carcinoma (HGSOC) and low-grade serous ovarian carcinoma (LGSOC) based on a two-tier system that considers nuclear atypia and mitotic rate^1,2^. LGSOC is relatively rare, comprising only 5–10% of all EOC cases^3^, and has distinct clinical, molecular, and epidemiological features compared to HGSOC. LGSOC tends to be well differentiated, detected at an earlier age, and is slow growing^4–8^, yet the response rates are much lower than HGSOC^7–9^. The genetic landscape also differs, for example, *TP53* mutation is prevalent in HGSOC, while *KRAS*, *BRAF*, and *ERBB2* alterations are more common in LGSOC^10^, as well as estrogen receptor (ER) positivity. Historically, there has been no clear distinction in treatment recommendations and management of LGSOC and HGSOC, as most clinical studies included both histologic types, and treatment regimens for LGSOC were extrapolated from HGSOC^9^. Although HGSOC has high rates (70–80%) of partial or complete response to standard-of-care paclitaxel and carboplatin chemotherapy, the poor response of LGSOC^7^ suggests that treatment paradigms should be different between these two distinct subtypes. Recent phase II studies have established a role for targeted therapies such as hormone receptor agonists or MEK inhibitors in the treatment of recurrent LGSOC^11^. Given the general resistance of LGSOC to chemotherapy and unfavorable prognosis, new approaches are needed to identify effective therapies.

**Fig. 1:**
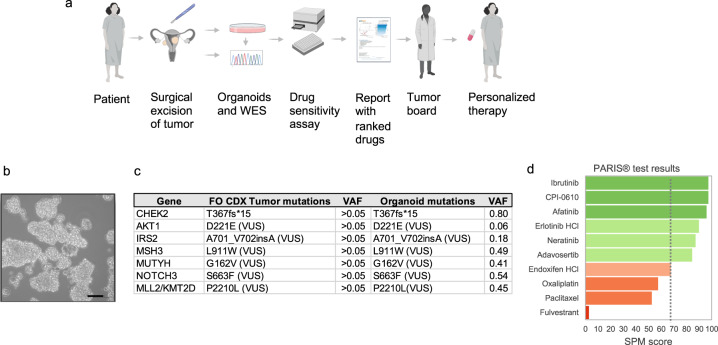
PARIS^®^ assay workflow including organoid generation from the fresh surgical specimen, genomic characterization, and report generation. **a** Schematic of the clinical integration of the PARIS^®^ drug sensitivity assay. WES: whole exome sequencing. Created with BioRender.com. **b** Brightfield photomicrograph of the patient’s cultured tumor organoids. Scale bar = 50 μm. **c** Mutational landscape of the primary tumor from Foundation One and all confirmed in tumor-derived organoids. The Foundation One (FO) report only reported mutations with >0.05 variant allele frequency (VAF) but did not report the actual value for each gene. ND not detected. **d** Table of top-scoring drugs in green from the PARIS® assay. In red are classes of drugs that the patient took prior to the PARIS^®^ test. The results indicating oxaliplatin, taxanes, and ER targeting drugs showed no to borderline (dotted line) sensitivity.

Patient-derived tumor organoids (PDTOs) have recently been developed as a model system to enable direct functional interrogation of a given patient’s tumor cells^12^. PDTOs retain histological and biological features and somatic genomic alterations from the originating tumor^13^ but also share the entire germline genomic profile, as well as any exposure or treatment history, all of which can affect drug sensitivity and response to therapy. Controlling for these variables could, in theory, enhance the predictive accuracy of patient-derived organoid models relative to other cancer models that are genetically unrelated to any given patient. The PARIS^®^ test is a CLIA-certified first-in-class high-throughput drug sensitivity assay that employs organoids cultured directly from solid tumors to test drugs or drug combinations in real-time for their potential efficacy^13,14^ (Fig. 1a). Because the assay is CLIA certified it can be used by physicians to inform treatment options or to guide clinical studies. Here, we describe a case report of a patient with LGSOC who progressed despite multiple rounds of standard-of-care treatments and two surgeries, leaving no therapeutic options. Tumor organoids were derived from the second surgical resection and subjected to drug sensitivity testing. The results showed exceptional sensitivity to the BTK inhibitor ibrutinib. Although ibrutinib is not FDA-approved for the treatment of ovarian cancer, the tumor board and oncologist recommended and requested its off-label use for this case. Following daily ibrutinib as a monotherapy, the patient exhibited a dramatic and prolonged clinical response resulting in stable disease for greater than 65 weeks. To our knowledge, this is the first report indicating the use of ibrutinib, an FDA-approved drug for lymphoma malignancies^15^ to treat chemotherapy-resistant ovarian cancer resulting in clinical benefit.

## Results

### Case history

**Fig. 2:**
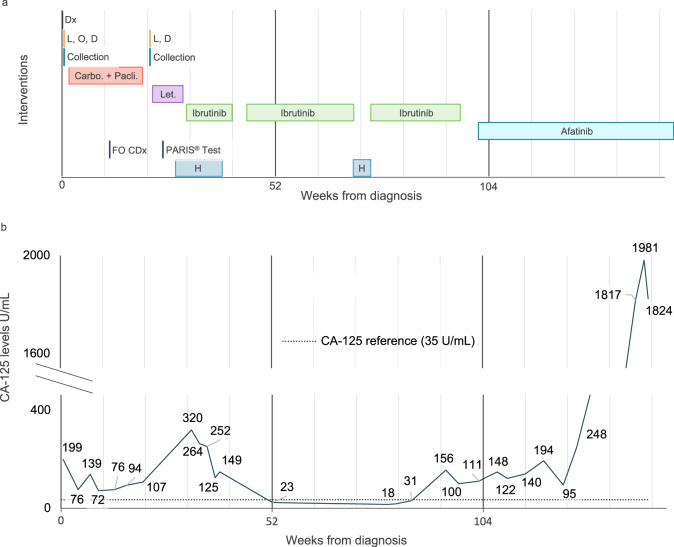
Clinical timeline of the patient and CA-125 levels. **a** Interventions. Dx diagnosis. L, O, D exploratory laparotomy, partial omentectomy, debulking (unsuccessful), L, D exploratory laparotomy, debulking (unsuccessful), FO CDx Foundation One test, H hospitalization. **b** CA-125 levels during the disease and treatment course.

A 52-year-old Gulf War veteran with a history of prior hysterectomy for fibroids presented with constipation and intermittent diarrhea thought to be related to irritable bowel syndrome (IBS). She was treated for IBS for 9 months until she presented to the emergency department with increased abdominal pain, distension, and constipation. A CT scan showed an 11cm complex adnexal mass, peritoneal carcinomatosis, and pericapsular implants in the liver with a small volume ascites (Fig. 1a). Surgery was recommended and the patient underwent an exploratory laparotomy, lysis of adhesions, partial omentectomy, and partial resection of the abdominal wall. Debulking was unsuccessful due to extensive intra-abdominal disease with omentum and anterior abdominal wall involvement. At the time of this surgery, the patient’s CA-125 level was 199 U/mL (Fig. 2b).

### Tumor stage, pathology, and genomics

The histological diagnosis from the debulking surgery was LGSOC, stage IIIC. Immunohistochemistry studies on a sample of omentum tissue collected during this initial surgery revealed diffuse PAX8 positivity, consistent with EOC. Further companion diagnostic testing by Foundation One, which interrogated 324 cancer genes including TP53, BRCA1, and BRCA2, revealed a somatic pathogenic alteration in CHEK2 (T367fs*15). Several variants of unknown significance (VUS) were also reported, namely AKT1 D221E, IRS2 A701_V702insA, MLL2 P2210L, MSH3 L911W, MUTYH G162V, and NOTCH3 S663F (Fig. 1c).

### Initial treatment

Following her diagnosis, the patient completed 6 cycles of paclitaxel (175 mg/m^2^) and carboplatin (AUC 6 IV) every 3 weeks in the adjuvant setting. Her CA-125 dropped to a nadir of 72 U/mL during treatment but rose to 94 U/mL during the sixth cycle (Fig. 2b). As her CA-125 level was steadily increasing, the patient underwent a second attempt at debulking surgery which was suboptimal due to significant bowel adhesive disease and with little evidence of response to chemotherapy.

### Tumor organoid-based drug testing

Based on the poor response to the standard of care and deteriorating conditions of the patient, the oncologist ordered the PARIS^®^ test to assess candidate drugs for their potential efficacy. On the day of the surgery, a sample of tumor tissue taken from the omentum was sent to SEngine’s CLIA laboratory. The sample had >70% tumor cell viability and was expanded as a 3D organoid culture for the drug screening assay. To confirm the tumor organoids represented the original tumor, genomic analysis of the organoid culture was performed using a CLIA-certified whole exome sequencing (WES) pipeline. Figure 1c shows confirmation of all seven mutations between the primary tumor and the organoid culture.

Drug screening was performed with a custom panel of 42 small molecule drugs selected based on standard-of-care chemotherapies, drugs targeting common cancer genes and pathways for LGSOC, and the genomic profile of the patient’s tumor, which included a CHEK2 mutation and several VUSs (Fig. 1c). The custom panel contained 34 targeted agents and 8 chemotherapies (fluorouracil, gemcitabine, methotrexate, oxaliplatin, paclitaxel, vinorelbine tartrate, SN-38, and doxorubicin HCl) (Supplementary Table 1). The drugs were ranked from the most effective to the least with a proprietary score report (SPM 100 to 1, with 100 to 70 considered active drugs). A report describing these results was sent to the treating oncologist 23 days after the sample was received (Fig. 1d). Of the eight chemotherapies tested, only gemcitabine had a low response (Supplementary Table 1) while the rest, including paclitaxel and oxaliplatin had no activity, consistent with her poor response to this class of agents. Carboplatin was not included as it was inactive in this drug library. In contrast to the resistance of the tumor organoids to chemotherapy drugs, multiple targeted agents showed good to exceptional activity, including the BTK inhibitor, ibrutinib (SPM score 97.3) and the EGFR inhibitor, afatinib (SPM score 95.9). Neratinib and erlotinib, both targeting the EGF receptor, were also active (SPM score 87.1 and 89.8, respectively) suggesting this patient’s tumor may be dependent on BTK and/or EGFR signaling. In addition, the experimental BET inhibitor CPI-0610 demonstrated an exceptional response (SPM score 97.3), and the WEE1 inhibitor adavosertib a moderate response (SPM score 84).

### Post-PARIS^®^ test treatments

Following the second surgery, the patient was treated with the aromatase inhibitor letrozole (2.5 mg p.o., daily) for 2 months, which is a standard second-line therapy for recurrent LGSOC. During this course of treatment, she was admitted to the hospital once for post-operative ileus and twice for malignant small bowel obstruction. A CT scan revealed omental caking indicative of chronic low-grade malignant bowel obstruction and a venting G-tube was placed.

### Ibrutinib monotherapy results in biomarker reduction, symptom improvement, and prolonged tumor control

Following a review of the PARIS^®^ test results and evaluation by an interdisciplinary molecular tumor board at the University of Washington, the treating oncologist elected to start treatment with ibrutinib (420 mg p.o., daily) as monotherapy. After four weeks of ibrutinib treatment, the patient’s CA-125 level decreased from 252 to 125 U/mL (Fig. 2b). She initially experienced anemia possibly linked to ibrutinib and was admitted several times for acute abdominal pain related to small bowel obstruction and carcinomatosis. As her pain was only able to be controlled with fentanyl patient-controlled-analgesia (PCA) and she was unable to eat, she elected to enter home hospice care. At this time, her disease was deemed end-stage with an expectation of weeks to months to live. She paused all treatment for one month and then restarted ibrutinib. Following the resumption of ibrutinib, her abdominal pain and bowel movements stabilized, and her CA-125 level decreased over the next 12 weeks from 149 to 23 U/mL. The patient showed continued improvement, with the ability to tolerate solid foods and bowel movements. After another 25 weeks, her CA-125 level had reduced further to 19.9 U/mL and a CT scan showed a slight improvement in carcinomatosis indicative of stable disease. During this time her performance status improved from ECOG 3 to ECOG 1 and her quality of life had improved so much that she was able to travel out of state and transition off PCA and opioid medication. The patient had a brief hold of ibrutinib for a several-week hospitalization due to line sepsis but restarted shortly thereafter.

After 65 weeks of stable disease on ibrutinib the patient’s CA-125 level began to rise, oscillating between 100 to ~150 U/mL (Fig. 2b). Afatinib, an FDA-approved EGFR inhibitor, was the third highest-scoring drug in the PARIS assay (Fig. 1d) and in consultation with the tumor board it was decided to put the patient on afatinib (150 mg p.o., daily) as monotherapy, since the second highest-scoring drug was experimental and not available for this patient. While on afatinib, her CA-125 levels remained stable in the low 100s for an additional 24 weeks and the patient had no hospitalizations. However, her hemoglobin levels frequently dropped below 8, requiring transfusions every 3–4 weeks from week 24 to 44. Because of the anemia and the rising levels of CA-125 to 1837 U/ml, afatinib was discontinued. The patient is currently taking erlotinib (150 mg p.o., daily), another EGFR inhibitor that was also among the top-scoring drugs in the PARIS assay (Fig. 1d). The patient’s Hb levels are currently at 9.1. In total, since starting ibrutinib and then continuing on afatinib, this patient has gone from hospice care with an inability to eat and an ECOG score of 3 and requiring opioids to control pain to an ECOG score of 1 with cessation of opioids and >20 months of stable disease.

## Discussion

Despite different biologic features and genetic profiles, the treatment of LGSOC is not substantially different from that of HGSOC. The relatively poor prognosis and early age at diagnosis of LGSOC highlight the need for new strategies to address this disease. Here we report the successful application of a tumor organoid-based drug sensitivity assay to identify several effective targeted therapies for an LGSOC patient who had exhausted treatment options and for whom genomic testing with Foundation One was unable to provide clear guidance for treatment. Tumor organoids were derived from a surgical excision in the course of a failed attempt at cytoreduction and subjected to drug testing. Consistent with the known chemoresistance of LGSOC, the organoids were nonresponsive to chemotherapeutic agents, including classes of drugs that the patient had taken. In contrast, drug testing identified several efficacious targeted drugs, with ibrutinib, CPI-0610, afatinib, erlotinib, and adavosertib as top-scoring drugs. Ibrutinib was selected following the tumor board review of the case and based on the PARIS^®^ assay results. It is noted that the patient was only able to obtain off-label ibrutinib because she was a veteran. Despite a terminal diagnosis, with multiple tumor-associated complications, ibrutinib monotherapy resulted in a dramatic clinical response and stable disease with normalization of CA-125 levels for ~15 months.

Ibrutinib is an orally administered small molecule inhibitor of BTK, an intracellular Src-family tyrosine kinase associated with B cell receptor signaling^16^. Ibrutinib is FDA-approved for the treatment of hematological diseases, specifically those of B-cell lineage, such as mantle cell lymphoma, chronic lymphocytic leukemia, small lymphocytic lymphoma, Waldenstrom’s macroglobulinemia (WM), and marginal zone lymphoma^15^. Ibrutinib forms covalent bonds with BTK at cysteine 481 near the ATP binding pocket, thus irreversibly blocking its kinase activity^17^. Ibrutinib has also been reported to have off-target activity against the ERBB/EGFR family, TEX family, and other tyrosine kinases^18^.

High BTK expression was associated with reduced overall survival in a cohort of 50 ovarian cancer patients and the combination of cisplatin and ibrutinib demonstrated synergy in two ovarian cancer cell lines^19^. Ongoing Phase I/II clinical trials are investigating the activity of ibrutinib in combination with durvalumab (NCT02403271), trastuzumab (NCT03379428), or nivolumab (NCT03525925) in solid tumors^20,21^. However, to our knowledge, ibrutinib has not been investigated as a monotherapy in ovarian cancer. There is one anecdotal report of a patient with chronic lymphocytic leukemia who also had LGSOC and who demonstrated a reduction of CA-125 levels following ibrutinib therapy^22^. Additional PARIS^®^ test results across a larger cohort of ovarian cancer patients indicate that ibrutinib could be of clinical utility for >10% of ovarian cancer patients, including those with HGSOC^23^ (manuscript in preparation).

In addition to ibrutinib, multiple EGFR-targeted agents including afatinib, neratinib, and erlotinib were identified as top-scoring drugs. After 65 weeks on ibrutinib, the patient started to progress and switched to afatinib also as monotherapy and had clinically stable disease for >44 additional weeks. Due to anemia and rising CA-125 levels, the therapy was switched to erlotinib and the patient is currently being evaluated for response. In total, since starting ibrutinib and then continuing on afatinib, this patient has had >24 months of stable disease. In comparison, Gershenson et al. reported a median progression-free survival of 7.2 months for patients with recurrent low-grade serous ovarian cancer^11^.

For this patient, genomic testing did not identify alterations that could readily explain either BTK or EGFR inhibitor sensitivity, other than a VUS in NOTCH3 which could conceivably act as a deactivating mutation and thereby increase sensitivity to EGFR targeted therapies^24^. In addition, the patient’s tumor had mutations in genes that are associated with genetic instability, including a CHEK2 mutation and VUSs in MSH3, and MUTYH. Drug screening did not reveal sensitivity to doxorubicin, platinum drugs, PARP inhibitors, or ATR inhibitors, suggesting these mutations in the context of this patient’s tumor biology and genetic landscape, did not confer sensitivity to these DNA damaging or DNA damage response (DDR) targeting drugs. This finding is concordant with a previous report that CHEK2 pathogenic mutations do not confer PARP inhibitor sensitivity^25^. The patient’s tumor also carried VUSs in the AKT/mTOR pathway genes AKT1 and IRS2. However, the organoid cultures were not sensitive to drugs that targeted these pathways. Collectively, these results illustrate the utility of ex vivo functional testing to probe the phenotypic consequences of cancer-associated mutations and VUSs. Importantly, phenotypic testing is performed in the identical tumor cells in which these mutations arose, thus preserving the relevant genetic, cellular, and clinical context, all of which can modify genotype:phenotype associations.

This patient’s tumor organoids also showed an exceptional response to the investigational BET inhibitor CPI-0610 and a good response to the WEE1 inhibitor adavosertib, suggesting additional targetable vulnerabilities. We previously demonstrated the sensitivity of ovarian clear cell carcinoma-derived organoids to the BET inhibitor CPI-0610^26^. Recently, adavosertib as monotherapy showed promising clinical activity in refractory solid tumors including ovarian cancer^27^. These agents were not prioritized for treatment because they are not FDA-approved.

This study, as well as results obtained across a large cohort of patients with solid tumors^23,28–30^ shows the clinical utility of organoid-based drug sensitivity testing to identify personalized and actionable treatments. Because the PARIS^®^ assay tests a broad menu of oncology drugs it increases the likelihood of identifying actionable therapies, especially in cases where genomic testing or other biomarkers are not informative.

## Methods

### Sample and patient intake process

Oncologists order the PARIS^®^ test by filling out a requisition form provided by SEngine Precision Medicine. Patients are contacted for optional consent to the IRB research protocol to enable clinical research and the use of residual material for research. Authorization for medical records is also an optional request to enable clinical research. This patient gave written consent to SEngine Precision Medicine to obtain original medical data and publish results.

### The PARIS^®^ assay

Briefly, organoids are established from surgical excisions, body fluids such as ascites, or biopsies, following CLIA-certified standard operating procedures. As soon as a >70% pure organoid culture is obtained, which for this patient was at 7 days, they are subjected to drug screening. A custom panel of 42 drugs was selected for this patient from a library of >200 oncology agents validated for activity. The SEngine drug library includes FDA-approved and experimental oncology drugs, chemotherapeutics, hormone antagonists, and small-molecule inhibitors. We performed 6-point dose–response assays, calibrated for each drug to cover the Cmax values, to ensure each drug is tested at a clinically relevant concentration. Drug concentrations ranged from 10 μmol/L to 33 pmol/L, depending on the individual drug properties. The patient’s organoid-based drug sensitivity was measured through a series of standard drug response metrics (IC50 and area under the curve—AUC) as well as proprietary algorithms. Drugs receive an SPM score from 100 to 1, with 100 being the most effective. Drugs that score below 70 are considered not effective for that patient. A CLIA-certified test report with these results was sent to the oncologist with a 23-day turnaround time from receipt of the specimen. Additional background can be found in pre-clinical research papers leading up to the PARIS^®^ assay^13,23,28–32^.

### Whole-exome sequencing

Next-generation CLIA-certified whole exome sequencing was performed by Fulgent Genetics. Genomic DNA was extracted from patient-derived tumor organoids and assessed for quality using a Bioanalyzer (Agilent Technologies). Target enrichment was then performed using the xGen Exome Research Panel v2 (Integrated DNA Technologies) followed by sequencing library preparation using the KAPA HyperPlus Library Prep kit. Indexed libraries were pooled for sequencing on multiple lanes on HiSeq (Illumina) or NovaSeq (Illumina) instruments to generate 150 bp paired-end reads at a read depth sufficient to obtain an average coverage of 300×.

### Computational analysis

An in-house pipeline that follows GATK best practices for somatic short variant and indel discovery (Broad Institute) was developed to process whole exome sequencing data. Briefly, short reads were mapped to the human NCBI Build 38 reference (hg38) using Burrows–Wheeler Aligner (BWA). Any potential PCR duplicates, ambiguous reads, inconsistent read pairs, or unmatched reads were excluded. Only unique reads that mapped in consistent read pairs (with proper insert size and orientation) were included for further analysis. Base substitutions and indels were called using five variant callers: FreeBayes 1.1.0^33^, GATK3, SAMtools v1.7^34^, VarScan2 v2.4.2^35^, and VarDict v1.5.1^36^. SnpEff v4.2^37^ and AnnoVar^38,39^ were used to annotate variants. To select high-confident somatic variants, the following filters were applied. Variants that were present in the Exome Aggregate Consortium (ExAC) reference dataset^40^ at a frequency ≥5% were considered germline variants and excluded from the analysis. Variants with a mapping quality of 20 or more were included. Variants that were called by at least two of the five variant callers were included. Variants with a mutation allele frequency <5% and read coverage <100× were excluded from the analysis.

### Reporting summary

Further information on research design is available in the Nature Research Reporting Summary linked to this article.

## Supplementary information

Supplementary Table 1

REPORTING SUMMARY

## Data Availability

The authors declare that data supporting the findings of this study are available within the article and its supplementary information files.

## References

[1] Gilks CB (2004). Subclassification of ovarian surface epithelial tumors based on correlation of histologic and molecular pathologic data. Int. J. Gynecol. Pathol..

[2] Malpica A (2004). Grading ovarian serous carcinoma using a two-tier system. Am. J. Surg. Pathol..

[3] Seidman JD (2004). The histologic type and stage distribution of ovarian carcinomas of surface epithelial origin. Int. J. Gynecol. Pathol..

[4] Kang JH (2020). Clinical factors associated with prognosis in low-grade serous ovarian carcinoma: experiences at two large academic institutions in Korea and Taiwan. Sci. Rep..

[5] Crispens MA (2002). Response and survival in patients with progressive or recurrent serous ovarian tumors of low malignant potential. Obstet. Gynecol..

[6] Vang R (2008). Subdividing ovarian and peritoneal serous carcinoma into moderately differentiated and poorly differentiated does not have biologic validity based on molecular genetic and in vitro drug resistance data. Am. J. Surg. Pathol..

[7] Schmeler KM (2008). Neoadjuvant chemotherapy for low-grade serous carcinoma of the ovary or peritoneum. Gynecol. Oncol..

[8] Gershenson DM (2009). Recurrent low-grade serous ovarian carcinoma is relatively chemoresistant. Gynecol. Oncol..

[9] Goulding EA, Simcock B, McLachlan J, van der Griend R, Sykes P (2020). Low-grade serous ovarian carcinoma: a comprehensive literature review. Aust. N. Z. J. Obstet. Gynaecol..

[10] Vang R, Shih IM, Kurman RJ (2009). Ovarian low-grade and high-grade serous carcinoma: pathogenesis, clinicopathologic and molecular biologic features, and diagnostic problems. Adv. Anat. Pathol..

[11] Gershenson DM (2022). Trametinib versus standard of care in patients with recurrent low-grade serous ovarian cancer (GOG 281/LOGS): an international, randomised, open-label, multicentre, phase 2/3 trial. Lancet.

[12] Wensink GE (2021). Patient-derived organoids as a predictive biomarker for treatment response in cancer patients. NPJ Precis. Oncol..

[13] Pauli C (2017). Personalized in vitro and in vivo cancer models to guide precision medicine. Cancer Discov..

[14] Narasimhan V (2020). Medium-throughput drug screening of patient-derived organoids from colorectal peritoneal metastases to direct personalized therapy. Clin. Cancer Res..

[15] Pharmacyclics Inc. Imbruvica® (ibrutinib) [package insert]. U.S. Food and Drug Administration Website. Accessed 1 Jan 2020.

[16] Pal Singh S, Dammeijer F, Hendriks RW (2018). Role of Bruton’s tyrosine kinase in B cells and malignancies. Mol. Cancer.

[17] Molina-Cerrillo J, Alonso-Gordoa T, Gajate P, Grande E (2017). Bruton’s tyrosine kinase (BTK) as a promising target in solid tumors. Cancer Treat. Rev..

[18] Berglöf A (2015). Targets for Ibrutinib beyond B cell malignancies. Scand. J. Immunol..

[19] Zucha MA (2015). Bruton’s tyrosine kinase (Btk) inhibitor ibrutinib suppresses stem-like traits in ovarian cancer. Oncotarget.

[20] Hong D (2019). A phase 1b/2 study of the bruton tyrosine kinase inhibitor ibrutinib and the PD-L1 inhibitor durvalumab in patients with pretreated solid tumors. Oncology.

[21] Metzler, J. M., Burla, L., Fink, D. & Imesch, P. Ibrutinib in gynecological malignancies and breast cancer: a systematic review. *Int. J. Mol. Sci.* **21**, 4154–4171 (2020). 10.3390/ijms21114154 PMC7312555 32532074

[22] Metzler, J. M., Fink, D. & Imesch, P. Ibrutinib could suppress CA-125 in ovarian cancer: a hypothesis. *Appl. Sci.* **11**, 222–228 (2021).

[23] Margossian A, Pollastro M (2021). A cancer organogram test as a guide for oncology treatments in SOLID tumors: an analysis of 628 tests in 419 patients. Presented at: ASCO Virtual Meeting; 2021. J. Clin. Oncol..

[24] Diluvio G (2018). NOTCH3 inactivation increases triple negative breast cancer sensitivity to gefitinib by promoting EGFR tyrosine dephosphorylation and its intracellular arrest. Oncogenesis.

[25] Abida W (2020). Non-BRCA DNA damage repair gene alterations and response to the PARP inhibitor rucaparib in metastatic castration-resistant prostate cancer: analysis from the Phase II TRITON2 Study. Clin. Cancer Res..

[26] Shigeta S (2021). Targeting BET proteins BRD2 and BRD3 in combination with PI3K-AKT inhibition as a therapeutic strategy for ovarian clear cell carcinoma. Mol. Cancer Ther..

[27] Fu, S. et al. Multicenter Phase II Trial of the WEE1 inhibitor adavosertib in refractory solid tumors harboring CCNE1 amplification. *J. Clin. Oncol*. JCO2200830 (2022). 10.1200/JCO.22.00830 PMC10489509 36469840

[28] Lui G (2021). Functional drug screening of organoids from ovarian cancer patients demonstrate clinical and genomic concordance and identifies novel therapeutic vulnerabilities. presented at: AACR Virtual Meeting; 2021. Cancer Res..

[29] Margossian A (2020). Organoid based functional test to predict personalized treatment in cholangiocarcinoma. presented at: AACR, Annual Meeting, SanDiego, CA; 2020. Cancer Res..

[30] Margossian, A. et al. Predictive value of a CLIA approved organoid based drug sensitivity test. presented at: ASCO Virtual Meeting; 2020; *J. Clin. Oncol.* **38** (suppl.), abstr 3630 (2020).

[31] Puca L (2018). Patient derived organoids to model rare prostate cancer phenotypes. Nat. Commun..

[32] Xu C (2018). Functional precision medicine identifies novel druggable targets and therapeutic options in head and neck cancer. Clin. Cancer Res..

[33] Garrison, E. & Marth, G. Haplotype-based variant detection from shortread sequencing. Preprint at 10.48550/arXiv.1207.3907.

[34] Li H (2009). The sequence alignment/Map format and SAMtools. Bioinformatics.

[35] Koboldt DC (2012). VarScan 2: somatic mutation and copy number alteration discovery in cancer by exome sequencing. Genome Res..

[36] Lai Z (2016). VarDict: a novel and versatile variant caller for next-generation sequencing in cancer research. Nucleic Acids Res..

[37] Cingolani P (2012). A program for annotating and predicting the effects of single nucleotide polymorphisms, SnpEff: SNPs in the genome of Drosophila melanogaster strain w1118; iso-2; iso-3. Fly (Austin).

[38] Wang K, Li M, Hakonarson H (2010). ANNOVAR: functional annotation of genetic variants from high-throughput sequencing data. Nucleic Acids Res..

[39] Yang H, Wang K (2015). Genomic variant annotation and prioritization with ANNOVAR and wANNOVAR. Nat. Protoc..

[40] Lek M (2016). Analysis of protein-coding genetic variation in 60,706 humans. Nature.

