# Supplementary material for: Extraordinary clinical response to ibrutinib in low-grade ovarian cancer guided by organoid drug testing
Source: NPJ Precis Oncol. 2023 May 18;7:45. doi: 10.1038/s41698-023-00379-8 (PMC10195827; doi:10.1038/s41698-023-00379-8)
Supplement: Supplementary file 1 — Supplementary Table 1 [file 41698_2023_379_MOESM1_ESM.pdf]

Supplementary Table 1

List of drugs tested and SPM score

Drug Name	Alternate Names	Target	SPM Score	CLIA Review Comments
Ibrutinib	Imbruvica, PCI-32765	BTK	97.3	Exceptional
CPI-0610	CPI0610	BET proteins - BRD2, BRD3, BRD4, BRDT	97.3	Exceptional
Afatinib	Gilotrif, BIBW-2992, Giotrif (EU), Tovok, Tomtovok,	EGFR, HER2	95.95	Exceptional
Fluorouracil	Adrucil, 5-FU	antimetabolite - thymidylate synthase inhibitor	93.75	False Positive
Erlotinib HCl	Tarceva, CP-358774, OSI-774, RG-1415, R-1415, NSC-718781, Ro50-8231	EGFR, ALK, JAK2 mutant (JAK2V617F)	89.86	Good
Gemcitabine HCl	Gemzar, Gemcitabine	antimetabolite - pyrimidine antagonist	88.75	Low-see mention in report
Neratinib	neratinib maleate, Nerlynx, PB-272, HKI-272, CAN-030	EGFR, HER1, HER2, HER4	87.16	Good
Adavosertib	AZD1775, MK-1775	Wee1	84.46	Good
Capivasertib	AZD-5363	Akt	80.41	Moderate
Trametinib	Mekinist, GSK-212, GSK-1120212, JTP-74057, TMT-212	MEK1, MEK2	74.32	Low
Axitinib	Inlyta, AG-013736, Inraita (Japan),	VEGFR1, VEGFR2, VEGFR3 KIT, PDGFR, ABL1(T315I)	72.97	Low
Methotrexate	Methotrexate sodium	folic acid antagonist	70	NOT Sensitive
Cobimetinib	Cotellic, RG-7421, GDC-0973, XL-518	MEK	69.59	NOT Sensitive
Endoxifen HCl	Endoxifen	ER alpha, ER beta	67.57	NOT Sensitive
Ceralasertib	AZD-6738,	ATR	62.16	NOT Sensitive

Ipatasertib	RG-7440, GDC-0068	pan-AKT	61.49	NOT Sensitive
Oxaliplatin	Eloxatine, Elplat	DNA synthesis inhibition	57.5	NOT Sensitive
Palbociclib HCl	Ibrance, PD-991, PD-0332991, PF-0332991, PF-332991, Paruboshikuribu (Japan), Iburansu (Japan), Palbociclib, PD0332991	CDK4, CDK6	57.43	NOT Sensitive
Alpelisib	Piqray, BYL-719	PI3K p110α	56.08	NOT Sensitive
Dasatinib Monohydrate	Sprycel, BMS-354825, NSC-732517, Dasatinib	BCR-ABL, SRC	54.05	NOT Sensitive
Paclitaxel	paclitaxel (albumin-bound), Abraxane, ABI-007, nab-paclitaxel, albumin-bound paclitaxel, ABX, paclitaxel albumin-stabilized nanoparticle, Abraxus	microtubule inhibitor, taxane	52.5	NOT Sensitive
Rucaparib Phosphate	Rubraca, PF-1367338, AG-14699, CO-338, Rucaparib	PARP1, PARP2, PARP3	51.35	NOT Sensitive
Venetoclax	Venclexta, ABT-199, GDC-0199, RG-7601, BCL-2i, Venclyxto,	BCL2	48.65	NOT Sensitive
Talazoparib	Talzenna, LT-673, BMN-673, MDV-3800	PARP	46.62	NOT Sensitive
Vinorelbine Tartrate	Navelbine, Vinorelbine	microtubule inhibitor	42.5	NOT Sensitive
Belinostat	Beleodaq, PXD101	HDAC	40.54	NOT Sensitive
Niraparib Tosylate	Zejula, MK-4827, ZL-2306, Niraparib	PARP1, PARP2	37.84	NOT Sensitive

Encorafenib	Braftovi, NVP-LGX818, LGX-818, ONO-7702	RAF kinase	36.49	NOT Sensitive
Midostaurin	Rydapt, PKC-412, CGP-41251	FLT3, PKCα, PKCβ, PKCγ, SYK, Flk-1, Akt, PKA, c-KIT, FGFR, SRC, PDFRβ, VEGFR1, VEGFR2	34.46	NOT Sensitive
Decitabine	Dacogen, E7373	panDNMT	33.78	NOT Sensitive
Erdafitinib	Balversa, JNJ-42756493, JNJ-493	FGFR	31.76	NOT Sensitive
Olaparib	Lynparza, MK-7339, AZD-2281, KU-0059436	PARP	31.08	NOT Sensitive
SN-38		topoisomerase I, active metabolite of irinotecan	28.75	NOT Sensitive
Abemaciclib	Verzenio, LY2835219, LY 2835219, LY-2835219, Verzenios (EU)	CDK4, CDK6	27.7	NOT Sensitive
Binimetinib	Mektovi, MEK-162, ARRY-162, ARRY-438162, ONO-7703, Balimek (EU)	MEK	23.65	NOT Sensitive
Alectinib	Alecensa, AF802, CH5424802, RO5424802, RG7853	ALK, HER2	22.97	NOT Sensitive
Cediranib	Recentin, Zemfirza	VEGFR	22.97	NOT Sensitive
Doxorubicin HCl	Doxil, pegylated liposomal doxorubicin hydrochloride, Caelyx (EU), Adriamycin	topoisomerase II inhibitor, anthracycline antitumor antibiotic	16.25	NOT Sensitive
Dabrafenib	Tafinlar, GSK-2118436, GSK-436, DRB-436, Dabrafenib mesylate	BRAF	14.19	NOT Sensitive

Everolimus	Afinitor, Votubia (EU), Afinitor DISPERZ (everolimus tablets for oral suspension), RAD-001	mTORC1	12.16	NOT Sensitive
Crizotinib	Xalkori, PF-2341066, Zakori	ALK, ROS1, MET	5.41	NOT Sensitive
Fulvestrant	Faslodex	selective estrogen receptor degrader	2.7	NOT Sensitive
